# Evidence of ERalpha and ERbeta selectivity and partial estrogen agonism in traditional Chinese medicine

**DOI:** 10.1186/1477-7827-12-97

**Published:** 2014-10-10

**Authors:** Dov Tiosano, Françoise Paris, Marina Grimaldi, Vera Georgescu, Nadège Servant, Zeev Hochberg, Patrick Balaguer, Charles Sultan

**Affiliations:** Pediatric Endocrinology, Meyer Children’s Hospital, Rambam Medical Center, Ruth and Bruce Rappaport Family Faculty of Medicine, Technion-Israel Institute of Technology, Haifa, 31096 Israel; Unité d’Endocrinologie Pédiatrique, CHU Arnaud de Villeneuve, Montpellier, France; Département d’Hormonologie, CHU Lapeyronie, et Université Montpellier 1, Montpellier, France; INSERM U896, IRCM, Montpellier, F-34298 France; Rappaport Family Institute for Research in the Medical Sciences, Haifa, 31096 Israel; Département de l’Information Médicale, CHU Montpellier, Montpellier, France

**Keywords:** Estrogen receptors, Traditional Chinese medicines

## Abstract

The use of complementary and alternative medicine and herbal products, especially traditional Chinese medicines, is progressively rising for both adults and children. This increased use is based on the popular belief that these medicines are safe and harmless. In this report, we describe the results of a bedside-to-bench study that involved a short-statured 4-year-old boy with deficiencies in growth hormone, thyroid stimulating hormone, and adrenocorticotropic hormone due to an ectopic posterior pituitary gland and invisible pituitary stalk. Although the boy was given replacement therapy with hydrocortisone and L-thyroxin, the parents refused to treat him with growth hormone and consulted a naturopath who prescribed a traditional Chinese medicine (TCM) to stimulate the boy’s growth. From the age of 20 months, the child’s growth was regularly monitored while he was being treated with hydrocortisone, thyroxin, and the TCM. Over a 36-month period, the child’s growth velocity accelerated (3 cm/year to 8 cm/year), his height increment substantially increased (-2 SD to -0.8 SD), and his bones matured. In the laboratory investigation, estrogen receptor (ER)alpha and ERbeta reporter cell lines were used to characterize the estrogenic activity of the TCM medicine and its 18 components, and the results established that the medicine and some of its components have estrogen receptor ERalpha and ERbeta selectivity and partial estrogen agonism. Partial estrogenic activity of the TCM was confirmed using whole-cell competitive binding, cell proliferation, and endogenous gene expression assays in the ERalpha-positive breast cancer cell lines. Although the presence of evidence is not always evidence of causality, we have concluded that this traditional Chinese medicine contains ingredients with estrogenic activity that can sustain bone growth and maturation without affecting other estrogen-dependent tissues.

## Background

Over the past few decades, the use of traditional Chinese medicines (TCMs) has become internationalized. Although most components in TCMs are derived from plants, some are also derived from animals. Irrespective of the source of their components, TCMs are generally perceived as being safe and easy to use [1–5].

Our interest in TCMs and their possible effect on growth began when a short-statured 4-year-old boy with multiple pituitary hormone deficiencies, whose parents refused to treat him with growth hormone, exhibited unexpected growth acceleration and rapid bone maturation without any untoward effects on the development of his breast and genitals. On questioning, the boy’s parents informed us that he was also being treated with a TCM which had been prescribed by a naturopath. This TCM comprised 18 different components, of which 16 were derived from plants.

Many plants have been identified as containing phytoestrogens, some of which have also been classified as selective ER modulators (SERMs) because they have been shown to inhibit or stimulate estrogen-like actions in a cell-type, tissue-specific, and dose-dependent manner [6]. We therefore hypothesized that the growth acceleration and bone-age advancement in our patient was due to the presence of phytoestrogens in the TCM, and that these phytoestrogens were acting as SERMs.

It is now well known that the human estrogen receptor (ER) exists as two subtypes, ERα and ERβ, and that the two subtypes are expressed in the human growth plate. Both subtypes are ligand-inducible transcription factors, and their activities are regulated by 17β-estradiol (E_2_), which is important for the growth and maintenance of a diverse range of tissues, such as the mammary gland, uterus, and bone, and physiological systems like the cardiovascular and central nervous systems. Specifically, estrogens have a well-defined critical role in bone maturation and longitudinal bone growth in boys and girls [7, 8], and clinical observations have confirmed the role of estrogens in regulating longitudinal bone growth and growth plate closure. Low estradiol levels were shown to enhance skeletal growth during early sexual maturation (the pubertal growth spurt), while high E_2_ levels during late puberty accelerated growth plate fusion and the cessation of longitudinal bone growth [7, 9]. In the absence of estrogens, as found in females and males with a mutation of the aromatase gene or in the presence of a mutation of the ERα gene, the duration of the pubertal growth spurt is prolonged and the affected individuals continue to grow slowly after sexual maturation because the growth plates do not fuse [8, 10, 11]. Furthermore, in some patients with growth hormone deficiency and advanced puberty, the pubertal growth spurt is observed, suggesting that estrogens may act directly on growth plate chondrocytes [12].

The effect of estrogens on target tissues depends on the balance between ERα and ERβ signaling [13] and, in phytoestrogens, on the varying degrees of selectivity for ERα and ERβ [14]. The expression of ERα and ERβ in the human growth plate, with no difference in the expression pattern over the course of puberty or between sexes, reinforces the idea that estrogens can influence growth plate chondrocytes [15].

It is known that ER contains two distinct and independent transcriptional activation functions, AF-1 and AF-2 [16]. The activity of AF-1, which is located at the N-terminal A/B region of the receptor, is constitutive, whereas the activity of AF-2, which is located at the COOH terminus in the hormone-binding domain, is estrogen-inducible. Full transcriptional activity of the ER is achieved through synergism between its AFs [17]. It has also been reported that AF-1 possesses strong ligand-independent activity in some cell types and on some promoters and that the partial agonist activity of the ER can be modulated by the activity of AF-1 [18]. The amino acids (aa) that are important for AF-1 activity are found in segments between aa 41 and 120–150 at the N-terminal A/B region of the receptor [19]. The molecular mechanism of estrogen action on growth plate closure has recently been clarified. Growth plate closure only occurs in mice following specific inactivation of AF-1 in the ERα. This finding indicates that growth plate closure is induced by functions of the ERα that do not require AF-1 and that ERα AF-1 opposes growth plate closure [20]. The role of ERβ in growth plate cartilage seems more questionable in males [21] since knocking out ERβ in male mice does not influence bone growth at any stage of their development [22–24].

In order to test our hypothesis, we undertook a study to determine whether any of the 18 components in the TCM possess ERα or ERβ activity. To this end, we determined the estrogenic potencies of the TCM and each of the 18 components using estrogen-responsive element (ERE)-based luciferase reporter ERα and the ERβ assays. Furthermore, we confirmed that the TCM had estrogenic activity using binding assays, endogenous E_2_-regulated genes expression and E2-regulated proliferation assays in an ERα - positive breast cancer MCF-7 cell line.

## Case report

The boy was born after an uneventful pregnancy at 37 weeks gestation with a birth weight of 2,910 grams and is the first child of healthy non-consanguineous parents. The clinical examination at birth revealed a severe micropenis (1 cm, <2.5 SDS) and bilateral undescended testes, and the results of clinical endocrinology examination revealed signs of neonatal hypoglycemia, central hypothyroidism, and growth hormone (GH) and adrenocorticotrophic hormone (ACTH) deficiencies (Table 1). Replacement therapy with hydrocortisone (8 mg/m^2^ BSA) and 50 μg/day L-thyroxin was initiated. The bone age of the child before the start of replacement therapy was 3 years and the results of a brain magnetic resonance imaging (MRI) study revealed an ectopic posterior pituitary gland and an invisible pituitary stalk. The parents refused the recommended GH replacement therapy and consulted a naturopath, who prescribed a TCM with 18 components, of which 16 were of plant origin and two were of animal origin (Table 2). In addition to the hydrocortisone and thyroxin, the child was treated with the TCM (2-4 mls/day per os) for 36 months, during which his growth was regularly monitored. Over this 36-month period, his growth velocity accelerated (3 cm/year to 8 cm/year), his SD score for height substantially increased (-2 to -0.8), and his bone age advanced to 7 years. No changes were noted in the development of his breasts or genitals.

**Table 1:** **Results of endocrine evaluation in the neonatal period and the growth hormone axis at different ages**

Neonatal period		Basal	Stimulated
			**/ ACTH**
	Cortisol (nmol/l)	20 (N > 300)	25 (N > 500)
	TSH (mU/l)	5 (N:0.7 – 15)	
	TT4 (μg/dl)	4 (N > 5)	
	GH (μU/ml) during hypoglycemia	8.2 (N > 20)	
**20 Months**			**/ Arginine**
	GH (μU/ml)	2.4	
			30’: 2.8 (N > 20)
			60’ 3
			90’ 2.8
			120’ 1.8
	IGF1 (nmol/l)	5.3 (N: 6.6-42.5)	
**52 Months**			

IGF1 (nmol/l 16 (N:5.7-26.4).

X’: time in minutes.

/ ACTH” serum cortisol level after stimulation testing with ACTH after 60 minutes.

TSH: thyroid-stimulating hormone.

TT4: total thyroxine.

/ Arginine: serum GH level after stimulation testing with arginine.

IGF-1: insulin-like growth factor 1.

**Table 2:** **The 18 components of the traditional Chinese medicine**

Botanical, zoological or pharmaceutical name	Pinyin transliteration	Amount in medicine (grams)	Identification code
*Polygonatum sibiricum* Redoute	huang jing	5	A
*Astragalus membranaceus* (Fisch.) Bge	huang qi	5	B
*Drynaria fortunei* (Kunz) J. Sm	gu sui bu	5	C
*Boswellia carterii* Birdw.	ru xiang	5	D
*Cervus nippon* Temmiinck	lu rong	5	E
*Gallus gallus domesticus* Brisson	ji nei jin	5	F
*Morinda officinalis* How	bai ji li	5	G
*Ophiopogon japonicus* Ker-Gawl	mai men dong	5	H
*Commiphora myrrha* Engl.	mo yao	5	I
*Rehmannia glutinosa* (Gaertn.) Libosch	shu di	5	J
*Dioscorea opposita* Thunb.	shan yao	6	K
*Cornus officinalis* Sieb. et Zucc.	shan zhu yu	5	L
*Bupleurum chinense* D.C.	chai hu	4	M
*Alisma plantago-aquatica* L.var. *orientale* Samuels	ze xie	3	N
Massa Ferentata Medicinalis	shen qu	2	O
*Glycyrrhiza uralensis* Fischer	gan cao	10	P
*Poria cocos* (Schw.) Wolf	fu ling	5	Q
*Citrus reticulata* Blanco	chen pi	5	R

Sixteen of the components are of plant origin and two of the components, lu rong (E) and ji nei jin (F) are of animal origin. Massa fermenta medicinalis (O) is a non-standardized mixture of a fermented preparation of wheat flour and bran and the fresh aerial parts of medicinal herbs that may include xanthium, apricot kernel, artemesia, polygonum, and phaseolus.

## Methods

### Chemicals, reagents, and preparation of tinctures

Dulbecco’s modified Eagle medium: Nutrient Mixture F12 (DMEM F12), fetal calf serum (FCS) and geneticin, were purchased from Life Technologies Inc., Cergy-Pontoise, France. Luciferin was purchased from Promega, Charbonnières, France. E_2_, genistein, ferutinin, puromycin, and 3-[4,5-dimethyliazol-2-yl]-2,5-diphenyltetrazolium bromide (MTT) were purchased from Sigma-Aldrich, Inc., St. Louis, MO, USA.

Ethanol tinctures (35%) of each component in the TCM (A-R) (Table 2) were prepared in the following manner. After weighing, each component was first minced with a surgical scalpel and then dissolved in 70% ethanol: 30% sterile water at a solvent to plant ratio of 3:1 (v/w). Each solution was then steeped at room temperature for 3 weeks on a horizontal agitator, and then filtered through sterilized tulle. The filtrate was then diluted in sterile water at a ratio of 2:1 (v/v) in order to obtain a 35% ethanol tincture.

Stock solutions (10 nM in DMSO) of E_2_, genistein, and ferutinin were stored at -20°C. The TCM and the 18 tinctures were stored at 4°C. Before each assay, the stock solutions, the TCM, and the 18 tinctures were serially diluted using culture medium.

### Determination of ER Activity of the herbal medicine and its components

ERE-luciferase reporter assays were done in reporter HELN-ER cell lines [25] to investigate whether the TCM and any of its components have ER activity. Briefly, these cells were generated in two steps. The estrogen-responsive reporter gene was first stably transfected into HeLa cells, generating HELN cell line (named HELN for HeLa-ERE-Luciferase-Neo) and, in a second step, these HELN cells were transfected with ERα (amino acids 1-595), ΔA/B deleted domain ERα (ΔA/B-ERα and amino acids 179-595), ERβ (amino acids 1-530) and ΔA/B-ERβ (amino acids 143-530) plasmid constructs to obtain the HELN-ERα, -ΔA/B-ERα, -ERβ, and -ΔA/B-ERβ cell lines, respectively.

The HELN-ER cells were cultured in DMEM F12 without red phenol, which was supplemented with 5% dextran-coated charcoal-treated FCS (DCC-FCS), 1% antibiotic, 1 mg/ml G418, and 0.5 μg/ml puromycin in a 5% CO_2_ humidified atmosphere at 37°C. For all assays, the HELN cells were cultured in DMEM, which was supplemented with 5% FCS and 1% antibiotic, in a 5% CO_2_ humidified atmosphere at 37°C.

The TCM and its 18 components were first tested for non-specific modulation of luciferase expression on the HELN parental cell line, which contains the same reporter gene as HELN-ER cells but is devoid of ER. Except for component P, no toxicity or non-specific modulation was observed at concentrations lower than 1%. Then, the TCM and its components were tested for their ER activity. For this purpose, HELN and HELN-ER reporter cells (5 × 10^4^ cells/well) were seeded into a 96-well white opaque tissue culture plate (Greiner reference 655083). Eight hours after seeding, the cells were then exposed for 16 hours at 37°C to serial dilutions of the TCM and its 18 components (1% to 0.0001% of the methanol extract). At the end of the 16-hour incubation, the medium that contained the TCM or the tinctures was removed and replaced by test culture medium that contained 0.3 mM luciferin. At this concentration, luciferin diffuses into the cells, which generate a “glow-type” luminescent signal that is very stable for several hours. This luminescence was measured for 2 seconds in a MicroBeta Trilux luminometer (EGG Wallac, Turku, Finland). All assays at each concentration or dilution were done in quadruplicate, and three experiments were done in order to establish the response curves. The results are expressed as a percentage of maximum luciferase activity (100%), which was obtained in the presence of 10 nM E_2_. In order to obtain an indirect measure of the affinity for ERs of the TCM and its 18 components, the estrogenic potency or the concentration that yielded the half-maximal luciferase activity (EC_50_) of the TCM and its 18 components was determined.

### Whole-cell ERα and ERβ competitive binding assays

HELN-ERα and HELN-ERβ cells were seeded into each well (7 × 10^4^ cells/well) of a 96-well white opaque tissue culture plate with a clear bottom (Greiner reference 655098) and grown in the test culture medium for 24 hours in a 5% CO_2_ humidified atmosphere. At the end of the 24 hours, the cells were exposed to increasing dilutions of the TCM (1-0.0003%) in the presence of 0.3 nM [^3^H]-E_2_ (41.3 Ci/mmol specific activity) for 3 hours at 37°C. At the end of the incubation, the liquid in each well was aspirated and the cells were washed three times with 100 μl of cold phosphate buffered saline (PBS). Scintillation fluid (50 μl) (LS-6000-SC, Beckman-Coulter, Roissy, France) was added to each well, and the amount of [^3^H]-bound radioactivity in the mixture was determined using the MicroBeta Trilux luminometer (EGG Wallac, Turku, Finland). Non-specific binding was determined in the presence of 100 nM unlabeled E_2_. Specific binding was calculated by subtracting non-specific binding from total binding and was expressed as a percentage of the maximum ER binding (100%), which was obtained in absence of the herbal medicine. The IC_50_ value was defined as the dilution at which [^3^H]-E_2_ binding was 50%. Determinations were made in quadruplicate in three separate experiments. The absence of toxicity was determined by visual inspection.

### MCF-7 cell proliferation assay

MCF-7 cells were seeded into each well (10^3^ cells/well) of a 96-well culture plate and grown in the test culture medium for 24 hours at 37°C. After 24 hours, the cells were exposed for 6 days at 37°C in a 5% CO_2_ humidified atmosphere to increasing concentrations of E_2_ (10^-12^-10^-9^ M) and increasing dilutions of the TCM (1-0.001%). At the end of the incubation period, the medium in each well was removed and replaced by 100 μl of test culture medium that contained 0.5 mg/ml 3-(4,5-methylthiazol-2yl)-2,5-diphenyltetrazolium bromide (MTT). At the end of a 4-hour incubation at 37°C, the MTT-formazan-containing medium was gently removed, and the remaining MTT-formazan crystals were dissolved by adding 100 μl DMSO. After shaking, the cell number in each well was determined from the absorbance of the solution, which was measured with a microplate spectrophotometer at an absorbance of 540 nm. Wells that contained only the test culture medium and MTT were used to blank the plate reader. Determinations were made in quadruplicate, and the data are expressed as the average absorbance of four wells at each concentration or dilution in three separate experiments. The results are expressed as a percentage of maximum luciferase activity (100%), which was obtained the presence of 10 nM E_2_.

### E2-induced gene expression assays

The effect of the TCM on endogenous estrogen-regulated gene expression was assessed by determining the mRNA levels of GREB1, pS2, RIP140, RARα and PR in MCF-7 cells using RT-PCR. For this purpose, MCF-7 cells were treated for 24 hours with either 10 nM E_2_ or a 0.3% dilution of the TCM. At the end of the treatment, RNA was extracted from the cells using the RNeasy RNA isolation kit (Qiagen, Courtaboeuf, France). For RNA extractions, two independent cultures were performed per condition. Reverse transcription was done on 1 μg total RNA using random hexamers and SuperScript™ II reverse transcriptase (Invitrogen) in a reaction solution which was diluted ten times for amplification. The mRNA levels of GREB1, pS2, RIP140, RARα, and PR were quantified by RT-PCR using SYBR^®^ Green reagents in a LightCycler^®^ RT-PCR System (Roche SAS, Boulogne-Billancourt, France). The expression levels of each gene were normalized to that of the housekeeping 28S gene, and quantified in relative units using qBase^PLUS^ [26]. Determinations were made in duplicate.

### Data analysis

Data are reported as mean ± standard deviation or standard error of the mean. The response curves were fitted and analyzed using the sigmoid dose-response function of a computerized graphics and statistics software package (Graph-Pad Prism, version 4.0, 2003, Graph-Pad Software Inc., San Diego, CA, USA).

The dose-response curves were fitted using a four-parameter log-logistic model. Statistical comparisons between parameters of fitted curves (EC_50_ and maximal activity) were performed with the drc package in R 3.0.2 software (R Development Core Team 2013: [27]). One-way ANOVA and Tukey's multiple comparison tests were performed to compare the effect of TCM on gene expression of the estrogen receptor versus E2 and a control. Statistical significance was set at 5%.

## Results

**Figure 1:**
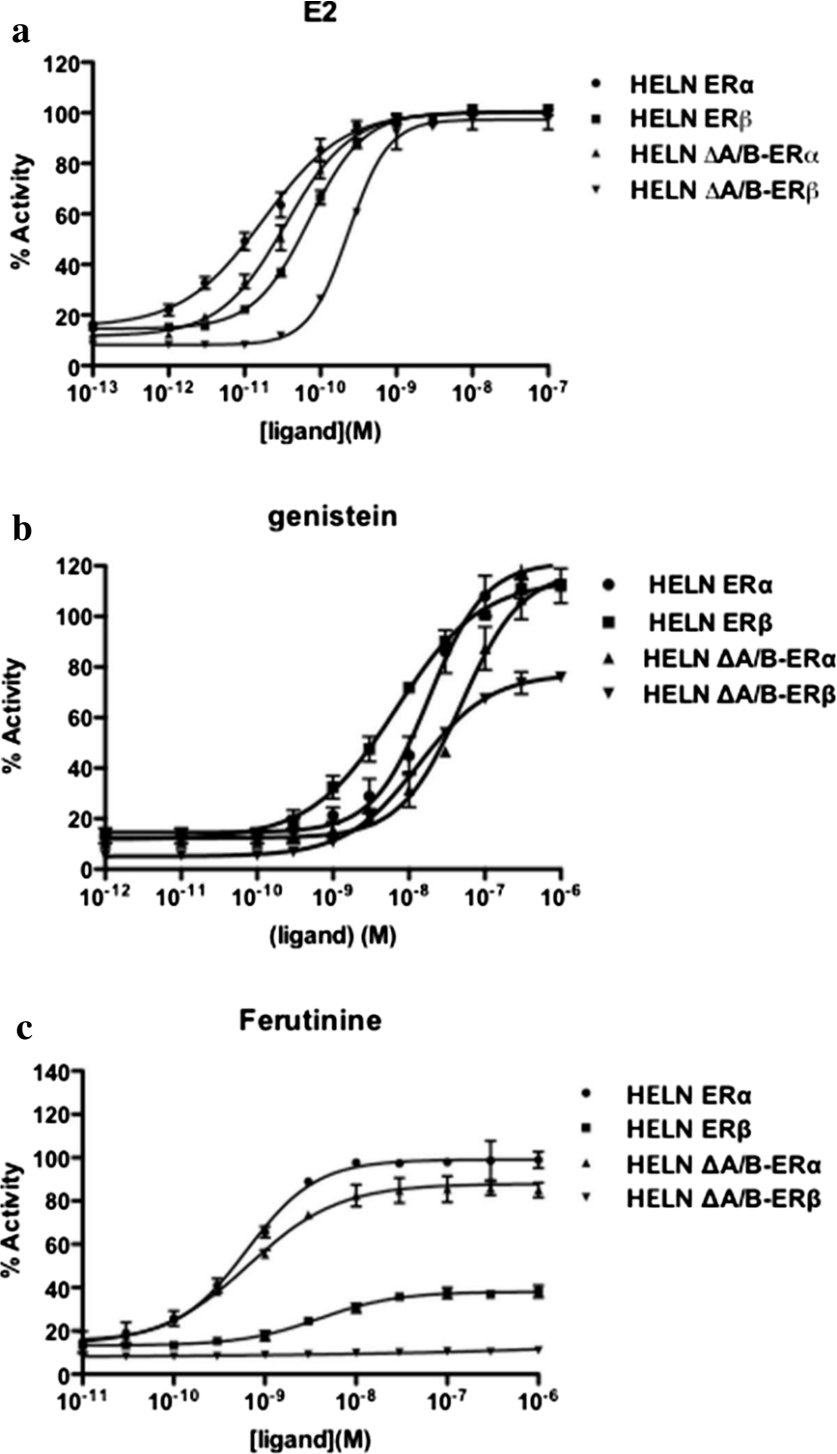
**The concentration-response curves to E**_**2**_ **(a), genistein (b), and ferutinin (c).** The curves were constructed by exposing HELN-ERα (ERα), HELN-ERβ (ERβ), HELN-ΔA/BERα (ΔAB-ERα), and HELN-ΔA/BERβ (ΔAB-ERβ) cells to increasing concentrations of E_2_, genistein, and ferutinin for 16 hours at 37°C in a 5% CO_2_ humidified atmosphere. The results are expressed as a percentage of maximum luciferase activity (100%), which was determined when the cells were exposed to 10^-8^M E_2_. Each value at each concentration in the response curves is the mean ± standard deviation from three separate experiments.

In order to evaluate the estrogenic activity of the TCM, we used previously established reporter cell lines [25, 28]. These cell lines, which expressed ERα, ERβ or these receptors deleted of their A/B region (ΔA/BERα, ΔA/BERβ), allowed characterization of ER specificity (between ERα and ERβ) and activity (antagonism, partial or full agonism). Before assessing the estrogenic activity of the herbal medicine, we used these cell lines to characterize two phytoestrogens, genistein and ferutinin, in comparison with the ER natural ligand, estradiol. Substantial differences in maximal activities and EC_50_s were detected in the concentration-response curves for E_2_ (Figure 1a) and the phytoestrogens genistein (Figure 1b) and ferutinin (Figure 1c). The EC_50_s for E_2_ in the HELN-ERα, HELN-ERβ, HELN-ΔA/BERα, and HELN-ΔA/BERβ cell lines were 0.017 nM, 0.068 nM, 0.034 nM, and 0.22 nM, respectively, and these values are in agreement with previously reported values [25, 28]. The EC_50_ of ERα for genistein was higher than that of ERβ (Figure 1b and Table 3), and this was due to its better affinity for ERβ than ERα [28]. Interestingly, deletion of the N-terminal A/B domain of ERβ had a partial effect on genistein’s transactivation efficacy (115.6 and 77.6% of maximal activity for ERβ and ΔA/BERβ, respectively) (Table 4), which confirms previously published findings that genistein is a partial ERβ agonist [29, 30]. Ferutinin appeared to be a full ERα agonist and a partial ERβ agonist (99.1 and 38.8% of maximal activity for ERα and ERβ, respectively) (Table 4). Moreover, ferutinin lost its partial activity when the N-terminal A/B domain of ERβ was deleted (Figure 1c).

**Table 3:** **EC**_**50**_ **of 17β estradiol (E**_**2**_ **); the phyotoestogen, genistein; the sesquiterpenoid, ferutinin; the traditional Chinese medicine (TCM); and the six selected components of the herbal medicine that were determined in the HELN-ERα, HELN-ERβ, HELN-ΔA/BERα, and HELN-ΔA/BERβ cell lines**

	ERα	ΔΑ/β ERα	ERβ	ΔΑ/β ERβ	ERα vs ERβ
					p-value
	***EC***_***50***_ ***(nM)***				
**E**_**2**_	0.017	0.034	0.068	0.22	
**Genistein**	18.8	49.4	6.1	18.8	
**Ferutinin**	0.64	0.65	3.9		
	***EC***_***50***_ ***(%)***				
**Traditional Chinese medicine**	0.035	0.055	0.015	0.035	**0.015**
**B**	0.2	0.25	0.055	0.15	**0.003**
**F**	0.2	0.2	0.03	0.1	**<0.001**
**J**	0.04	0.04	0.02	0.03	**NS**
**L**	0.3	0.3	0.4	0.4	**NS**
**P**	0.004	0.007	0.002	0.004	**NS**
**R**	0.07	0.08	0.035	0.06	**NS**

The EC_50_ values for E_2_, genistein, and ferutinin are expressed in nM, whereas those of the TCM and the components are expressed as percentages and correspond to dilutions of the TCM or components (1% = 100-fold dilution). Significant p-values corresponding to Student's t-test are reported (NS: not significant at the 5% significance level).

**Table 4:** **Maximal transcriptional activity of 17β estradiol (E**_**2**_ **); the phyotoestogen, genistein; the sesquiterpenoid, ferutinin; the traditional Chinese medicine (TCM); and the six selected components of the herbal medicine that were determined in the HELN-ERα, HELN-ERβ, HELN-ΔA/BERα, and HELN-ΔA/BERβ cell lines**

	ERα	ΔΑ/β ERα	ERα vs ΔΑ/β ERα	ERβ	ΔΑ/β ERβ	ERβ vs ΔΑ/β ERβ
	***Max luciferase activity (%)***		p-value	***Max luciferase activity (%)***		p-value
**E**_**2**_	100	100		100	100	
**Genistein**	121	117		115.9	77.6	
**Ferutinin**	99.1	87.9		38.8	12	
**Traditional Chinese medicine**	107.4	58	**0.014**	78	56	**<0.001**
**B**	80	39.6	**NS**	100	50	**0.021**
**F**	94	57	**0.002**	86.5	53	**<0.001**
**J**	78	29	**NS**	61	35	**NS**
**L**	69	23	**NS**	49	16	**NS**
**P**	111	70	**0.021**	92	73	**NS**
**R**	85	33	**0.004**	35	19	**NS**

Significant p-values corresponding to Student’s t-test are reported (NS: not significant at the 5% significance level).

We then tested the estrogenic activity of the TCM and its 18 components. The EC_50_s for ERα and ERβ and the maximal activity are summarized in Tables 3, 4 and 5. The EC_50_ for ERβ of the TCM was slightly lower than that of ERα (0.015% versus 0.035%) (p = 0.015). All the 18 components were found to have estrogenic activity and we were able to determine the EC_50_s for ERα and ERβ s for eight components, namely B, F, I, J, L, M, P, and R (Table 5). The EC_50_s for ERβ of the TCM and four of its components, I, J, P, and R, were slightly lower than those for ERα, but not significantly at the 5% significance level. The EC_50_s of two components for ERβ (B and F) were significantly lower than those for ERα (p = 0.003 and p < 0.001, respectively). The EC_50_s for ERα and ERβ of the remaining two components, L, and M, were very similar (Table 3).

**Table 5:** **The EC**_**50**_ **and maximal transcriptional activity of the traditional Chinese medicine (TCM) and its 18 components that were determined in the HELN-ERα and HELN-ERβ cell lines**

		ERα		ERβ	
		Max. luciferase activity %	EC _50_ %	Max. luciferase activity %	EC _50_ %
Traditional Chinese medicine		107.4	0.035	78	0.015
Huang Jing	A	48		51	
Huang Qi	B	80	0.2	100	0.055
Gu Sai Bu	C	34		23	
Ru Xiang	D	38		23	
Lu Rong	E	74		33	
Ji Nei Jin	F	94	0.2	86.5	0.03
Bai Ji Li	G	44		33	
Mai Men Dong	H	93		38	
Mo Yao	I	43	0.009	37	0.004
Shu Di	J	78	0.04	61	0.02
Shan yao	K	73		45	
Shan Zu Yu	L	69	0.3	49	0.4
Chai Hu	M	57	0.1	43	0.1
Ze Xie	N	33		20	
Shen Qu	O	100		61	
Gan Cao	P	111	0.004	92	0.002
Fu Ling	Q	108		45	
Chen Pi	R	85	0.07	35	0.035

The EC_50_ values of the herbal medicine and the 18 components are expressed as percentages and correspond to dilutions of the TCM or component (1% = 100-fold dilution). The percentage of maximum luciferase activity (100%) was determined when the cells were exposed to 10^-8^M E_2_. The complete identification code of the 18 herbal components is explained in Table 2.

**Figure 2:**
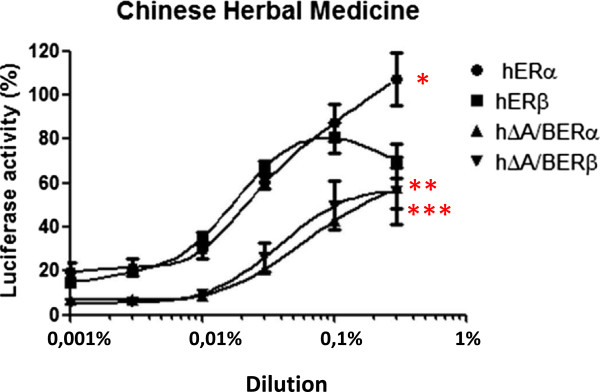
**The dilution-response curves to the traditional Chinese medicine (TCM).** The curves were constructed by exposing HELN-ERα (ERα), HELN-ERβ (ERβ), HELN-ΔA/BERα (ΔAB-ERα), and HELN-ΔA/BERβ (ΔAB-ERβ) cells to increasing dilutions of the TCM for 16 hours at 37°C in a 5% CO_2_ humidified atmosphere. The results are expressed as a percentage of maximum luciferase activity (100%), which was determined when the cells were exposed to 10^-8^M E_2_. Each value at each dilution in the response curves is the mean ± standard deviation from three separate experiments. p < 0.05: * = EC_50_ ERα vs ERβ, ** = Max. luciferase activity ERα vs ∆ A/B ERα, *** = Max luciferase activity ERβ vs ∆ A/B ERβ.

**Figure 3:**
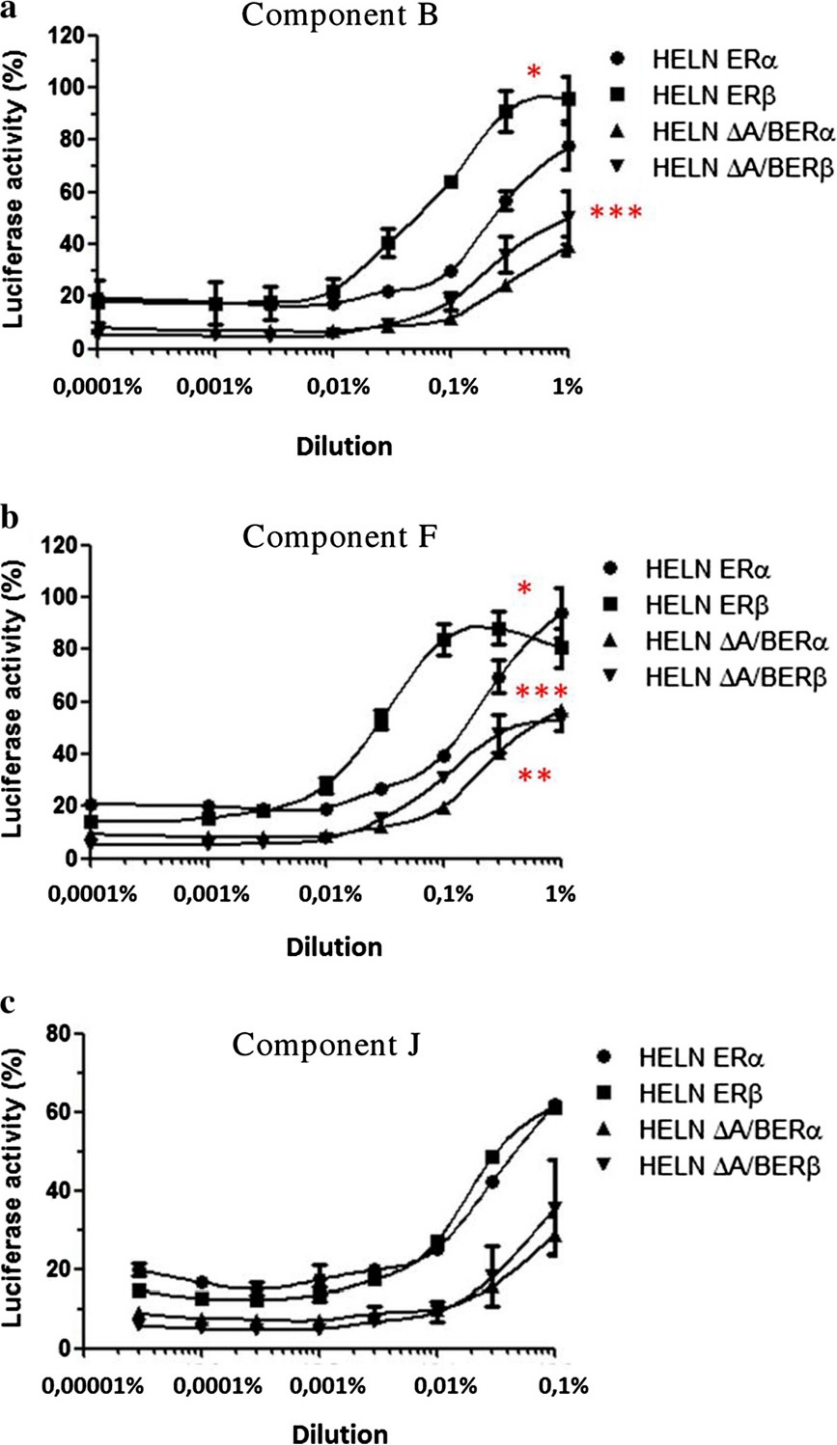
**The dilution-response curves to components B, F, J.** The curves were constructed by exposing HELN-ERα (ERα), HELN-ERβ (ERβ), HELN-DA/BERα (DAB-ERα), and HELN-DA/BERβ (DAB-ERβ) cells to increasing dilutions of ingredient B **(a)**, F **(b)**, J **(c)** of the TCM for 16 hours at 37°C in a 5% CO_2_ humidified atmosphere. The results are expressed as a percentage of maximum luciferase activity (100%), which was determined when the cells were exposed to 10^-8^M E_2_. Each value at each dilution in the response curves is the mean ± standard deviation from three separate experiments. p < 0.05: * = EC_50_ ERα vs ERβ, ** = Max. luciferase activity ERα vs ∆ A/B ERα, *** = Max luciferase activity ERβ vs ∆ A/B ERβ.

**Figure 4:**
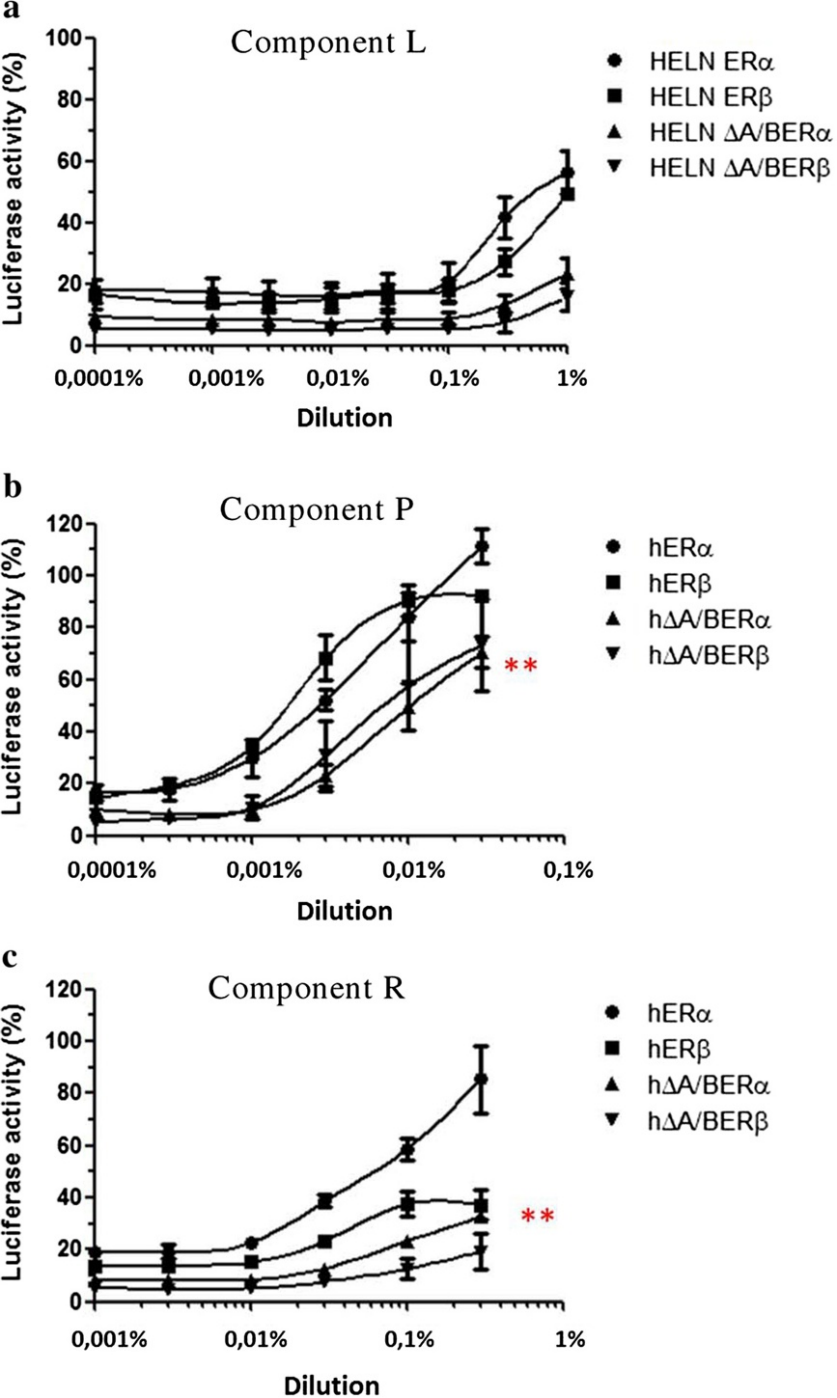
**The dilution-response curves to components L, P, R.** The curves were constructed by exposing HELN-ERα (ERα), HELN-ERβ (ERβ), HELN-DA/BERα (DAB-ERα), and HELN-DA/BERβ (DAB-ERβ) cells to increasing dilutions of ingredient L **(a)**, P **(b)**, R **(c)** of the TCM for 16 hours at 37°C in a 5% CO_2_ humidified atmosphere. The results are expressed as a percentage of maximum luciferase activity (100%), which was determined when the cells were exposed to 10^-8^M E_2_. Each value at each dilution in the response curves is the mean ± standard deviation from three separate experiments. p < 0.05: * = EC_50_ ERα vs ERβ, ** = Max. luciferase activity ERα vs ∆ A/B ERα, *** = Max luciferase activity ERβ vs ∆ A/B ERβ.

The HELN-ΔA/BERα and HELN-ΔA/BERβ cell lines were then used to further characterize the agonistic properties of the TCM and six of the eight components, which presented the maximal transcriptional activity on ERs (Table 4, Figures 2, 3 and 4). We found that the maximal activity values of the TCM and the six selected components in the HELN-ΔA/BERβ and HELN-ΔA/BERα cells were always lower than those found in the HELN-ERα and HELN-ERβ cells (Table 4). For example, we found that the maximal activity of the TCM in the HELN-ERα cells was 107.4% versus 58% (p = 0.014) in the HELN-ΔA/BERα cells. This finding was confirmed in HELN-ERβ and HELN-ΔA/BERβ cells: the maximal activity of the TCM in the HELN-ERβ cells was 78% versus 56% (p = 0.026) in the HELN-ΔA/BERβ cells. This result suggests that the TCM and the six selected components have partial ER agonist activity.

**Figure 5:**
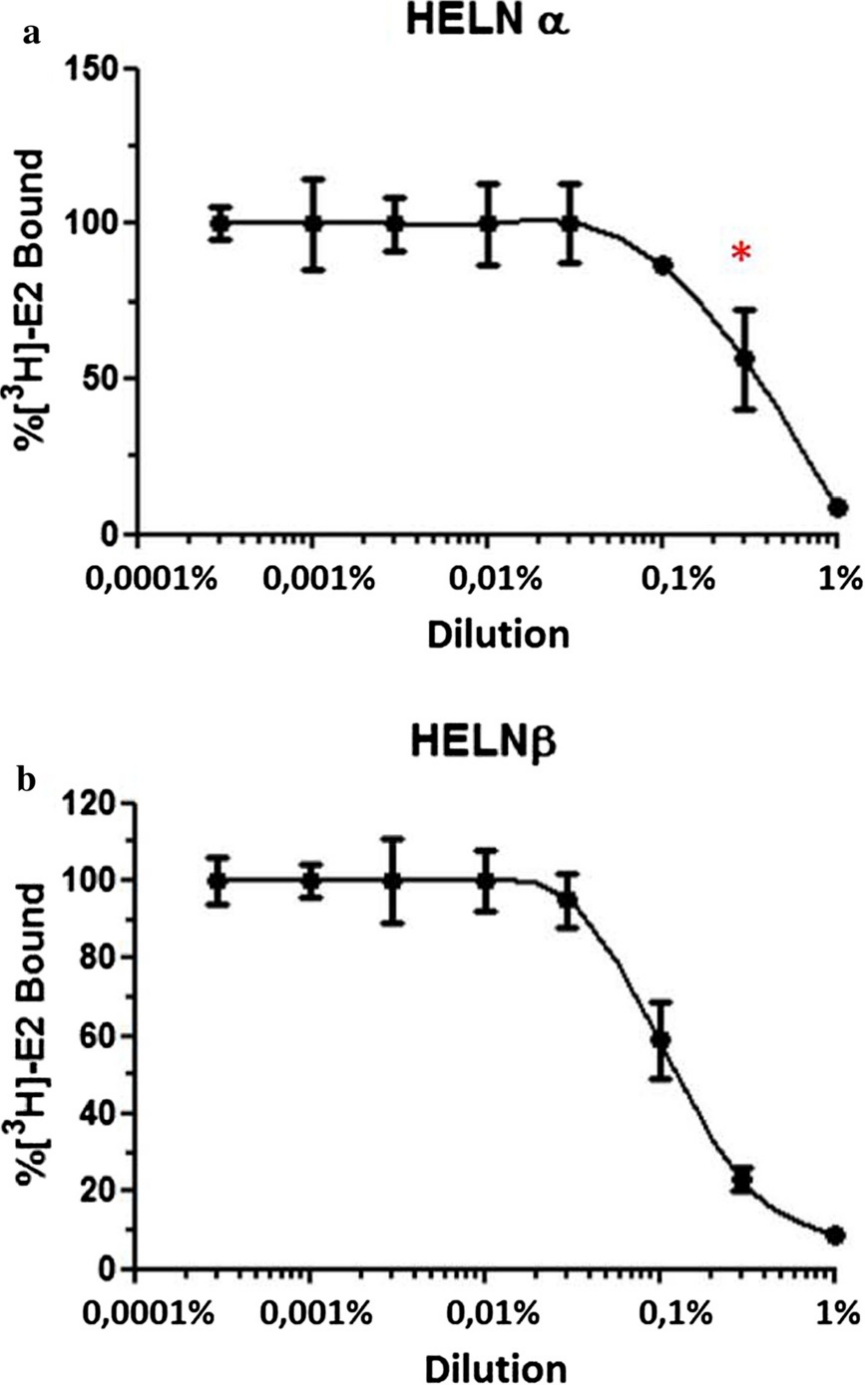
**The effect of the traditional herbal medicine (TCM) on E**_**2**_ **binding to estrogen receptor (ER)α and ERβ.** The competition binding curves for ERα **(a)** and ERβ **(b)** were generated by exposing HELN-ERα and HELN-ERβ cells, respectively, to increasing dilutions of the TCM (1%-0.0003%) in the presence of radiolabeled 17β-estradiol (E_2_) (0.3 nM [^3^H]-E_2_) for 3 hours at 37°C in a 5% CO_2_ humidified atmosphere. The amount of binding was expressed as a percentage of the maximum ER binding (100%), which was defined when the cells were exposed to 100 nM unlabeled E_2_. The IC_50_ value was defined as the dilution at which E_2_ binding was 50%. Each value at each dilution in the binding curves is the mean of duplicate determination ± standard deviation from three separate experiments. * = p < 0.05 for IC_50_ ERα vs ERβ.

We then characterized the estrogenic activity of the TCM. Whole-cell ER competitive binding assays using [^3^H] E2 binding to ERα and ERβ were then done in order to confirm that the ER activity of the TCM is achieved by binding to ERα and ERβ. The IC_50_ for ERβ of the TCM was 0.1% and this value was slightly lower than its IC_50_ for ERα, 0.3% (p < 0.05). This finding also confirms that the binding affinity for ERβ of the TCM was slightly greater than that for ERα (Figure 5).

**Figure 6:**
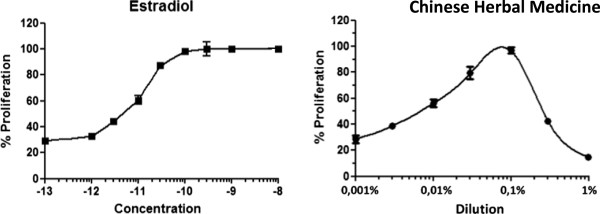
**The proliferative response of MELN cells to the traditional Chinese medicine (TCM).** The proliferation curves were generated by exposing HELN cells to increasing concentrations of E_2_ (10^-13^M – 10^-8^ M) and increasing dilutions of the TCM (1% - 0.001%) for 10 days, and then to the vital mitochondrial dye, 3-[4,5-dimethyliazol-2-yl]-2,5-diphenyltetrazolium bromide (MTT), for four hours at 37°C in a 5% CO_2_ humidified atmosphere. The data are expressed as a percentage of the maximum proliferation (100%), which was defined as the amount of proliferation when the cells were exposed to 10^-8^M 17β-estradiol. Each value at each concentration or dilution in the curves is the mean of quadruplicate determinations ± standard deviation from three separate experiments.

**Figure 7:**
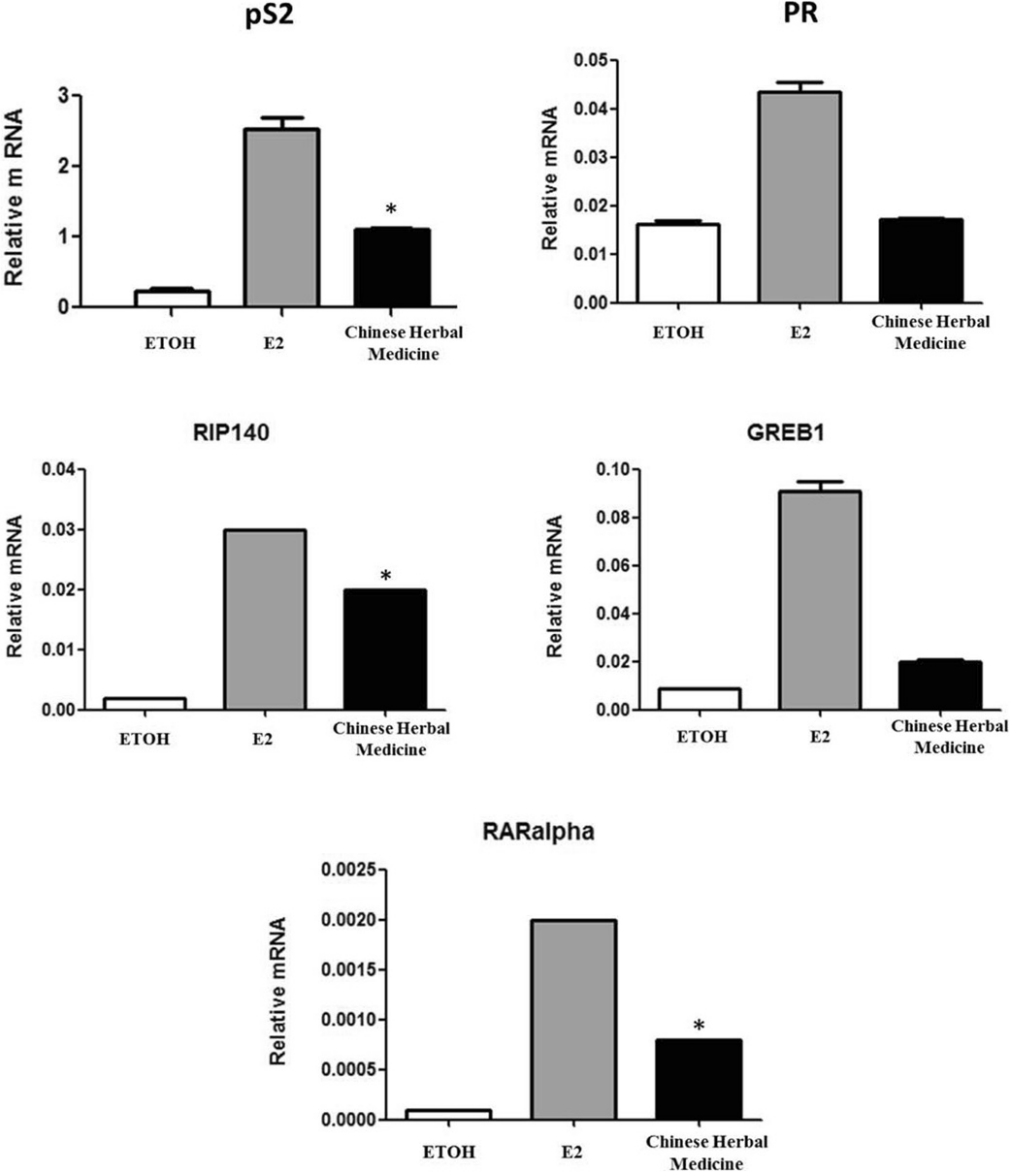
**The effect of the traditional Chinese medicine (TCM) on gene expression of the estrogen receptor.** MELN cells were treated for 24 hours at 37°C in a 5% CO_2_ humidified atmosphere with either 10^-8^M 17β-estradiol (E_2_) or a 0.3% dilution of the TCM. At the end of the incubation, the mRNA expression levels of five endogenous E_2_-regulated genes, pS2, the progesterone receptor (PR) , RIP140 , GREB1 , and RARα , were determined by quantitative real-time polymerase reaction. The expression levels of each gene were normalized to that of the housekeeping 28S gene, and quantified in relative units using qBase ^PLUS^[26]. Data are displayed as the average of two determinations ± standard error of the mean. * = p < 0.05 vs ETOH.

We then determined whether the TCM was able to activate endogenous E2-regulated genes in the ERα^+^ breast cancer cell line MCF-7. For HELN cell proliferation, we first found that the medicine acted as a full agonist on cell growth and that cell proliferation began to be activated at 0.003% (Figure 6). We also found that the TCM medicine was able to activate the expression of the endogenous E_2_-regulated genes, namely pS2, PR, RIP140, RARα, and GREB1 (Figure 7). This was statistically significant at the 5% significance level for pS2, RIP140 and RARα.

## Discussion

The billowing use of alternative medicines and in particular herbal products and TCMs by adults and children [31] is based on the popular belief that these products are “harmless.” Based on the knowledge that there are numerous estrogens with varying degrees of selectivity for ERα and ERβ in many TCMs [14], we hypothesized that the TCM, which was prescribed by a naturopath to a short-statured 4-year-old boy with multiple pituitary hormone deficiencies, would contain components with potent estrogen activity and would be responsible for the child's growth acceleration and bone maturation.

In order to test this hypothesis, we investigated the estrogenic activity of the TCM and its 18 components in a chemistry-focused study and a target-directed study. In the chemistry-focused study, we investigated the estrogenic activity of the TCM and its 18 components and found that the TCM and its components possess partial ERβ and ERα agonist activity with a slightly higher affinity for ERβ. In the target-directed study, we investigated the mechanism of action of the TCM constituents using reporter HELN-ERβ and ERα-positive cell lines and confirmed partial estrogenic activity using whole-cell competitive binding, cell proliferation, and endogenous gene expression assays. From these results, we have concluded that the TCM contains compounds that could be considered to be potential selective estrogen receptor modulators (SERMs) with specific agonist estrogenic activity.

A recent work demonstrated the stimulating action of a phyto-SERM, resveratrol, on growth plate cartilage [32]. This phyto-SERM displayed E_2_ antagonist activity for ERα with selected EREs and agonist activity for ERβ [33]. In rabbits, resveratrol improves both axial and appendicular bone growth without altering the serum insulin-like growth factor-1 (IGF-1) level [32]. Nevertheless, ERα and ERβ (activity) may also have a direct action on IGF-1 gene expression [34] and IGF-1-induced responses [35]. It is thus possible that the estrogenic actions of some of the TCM components influenced the serum IGF-1 levels in our study patient.

Did we prove this hypothesis in this bedside-to-bench study? We found that the TCM and some of its components have ERα and ERβ selectivity and partial estrogen agonism. Accordingly, we concluded that the complex estrogenic activity of this TCM was due to the presence of ingredients with estrogenic activity that are able to sustain bone growth and maturation without affecting other estrogen-dependent tissues. The well-known dominant negative effect of ERβ on ERα, as well as the partial and AF-1-dependent agonist activity of the TCM, may explain the absence of breast development in this patient [36].

## Conclusions

This study has several limitations. The first limitation is the dilution of the phytoestrogens in the various assays that were used to establish the pharmacological profile for the estrogenic activity of the TCM and the resultant circulating phytoestrogen levels in the patient. The TCM was taken orally by the patient, and we can assume that the serum and/or tissue concentrations of the TCM and its constituents would be lower than those in the TCM. Although we investigated a range of dilutions of the TCM and its constituents, we found that six of these components have partial estrogenic activity. The exact phytochemical and pharmacological profiles of the components are still unknown, and many drugs are converted to metabolites that retain the intrinsic activity of the parent drug. This lack of knowledge is a second limitation of our study. We are not aware of any studies in which a phytochemical profile of the ingredients in the TCM has been correlated with an in vitro and/or in vivo pharmacodynamic profile for phytoestogenic activity or a pharmacokinetic profile for the parent ingredients and their metabolites. The third limitation of our study is the quality and/or purity of the medicine’s components and the method of preparing the TCM. In this bedside-to-bench study, we found that the TCM and some of the components had varying estrogenic activity and potency over a large dilution range. Finally, we did not use cultured osteoblasts to investigate the estrogenic activity and mechanism of action of the TCM and its 18 components. Although it is tempting to conclude that the complex estrogenic activity of the TCM that was given to our patient accounted for unexpected growth acceleration and rapid bone maturation without any untoward effects on the development of his breast and genitals, the finding of accelerated growth velocity after ingestion of the TCM could be viewed as coincidental because the TCM is a non-standardized mixture of 18 components and causality cannot be unequivocally proved. Although the presence of evidence is not always evidence of causality, the challenge now is to identify those constituents in the TCM that may be therapeutically beneficial for growth-deficient children and then to develop them as therapeutic agents for these individuals.
